# Supplementary material for: Development of a computer-aided design software for the quantitative evaluation of aesthetic damage
Source: PLoS One. 2019 Dec 18;14(12):e0226322. doi: 10.1371/journal.pone.0226322 (PMC6919621; doi:10.1371/journal.pone.0226322)
Supplement: S1 Code site — 1. (ZIP) [file pone.0226322.s001.zip › [EESC_jr][Projeto]Relatorio_07_21/reports/templates_zip/gentelella-master/vendors/Flot/examples/index.html]

Flot Examples


## Flot Examples

Here are some examples for Flot, the Javascript charting library for jQuery:

### Basic Usage

- Basic example
- Different graph types and simple categories/textual data
- Setting various options and annotating a chart
- Updating graphs with AJAX and real-time updates

### Interactivity

- Turning series on/off
- Rectangular selection support and zooming and zooming with overview (both with selection plugin)
- Interacting with the data points
- Panning and zooming (with navigation plugin)
- Automatically redraw when window is resized (with resize plugin)

### Additional Features

- Using other symbols than circles for points (with symbol plugin)
- Plotting time series, visitors per day with zooming and weekends (with selection plugin) and time zone support
- Multiple axes and interacting with the axes
- Thresholding the data (with threshold plugin)
- Stacked charts (with stacking plugin)
- Using filled areas to plot percentiles (with fillbetween plugin)
- Tracking curves with crosshair (with crosshair plugin)
- Plotting prerendered images (with image plugin)
- Plotting error bars (with errorbars plugin)
- Pie charts (with pie plugin)
- Rendering text with canvas instead of HTML (with canvas plugin)

Copyright © 2007 - 2013 IOLA and Ole Laursen
